# Fabry Disease p.M290I Mutation is Related to Organ Involvement: A Case Report

**DOI:** 10.7759/cureus.14100

**Published:** 2021-03-25

**Authors:** Francisca Silva, Nicole Pestana, José Durães, Nuno Guimarães Rosa, Gil Silva

**Affiliations:** 1 Nephrology, Hospital Dr. Nélio Mendonça, Funchal, PRT; 2 Nephrology, Hospital Central do Funchal, Funchal, PRT

**Keywords:** genetic screening, x-linked genetic diseases, chronic renal failure, hereditary ventricular hypertrophy

## Abstract

Fabry disease (FD) is an X-linked hereditary disease. It results from mutations in the GLA gene, leading to deficient activity of the enzyme alpha-galactosidase A (α-Gal A) and progressive accumulation of undegraded glycosphingolipids in cell lysosomes. Enzyme replacement therapy (ERT) can improve the natural course of this disease, but an early diagnosis is crucial for a successful treatment.

We describe the case of a female diagnosed with chronic proteinuric kidney disease in the postpartum period. Despite receiving optimal medical treatment, the disease progressed and she started renal replacement therapy (RRT) with peritoneal dialysis (PD). Five years later, she was enrolled in a pilot screening study for FD, and the heterozygous mutation c.870G>C (p.Met290Ile; M290I) in exon six of the GLA gene was found. The family screening revealed the presence of this mutation in the patient’s father and daughter. The proband did not meet the criteria for a definitive FD diagnosis, but she remained under follow-up at our nephrology metabolic diseases consultation, as the mutation was described as pathogenic and associated with a classic FD phenotype. Later that same year, reassessment exams revealed a worsening left ventricle mass index (LVMi), a new ischemic cerebral lesion, and a substantial increase in serum globotriaosylsphingosine (LysoGb3) levels. These clinical changes led to a decision to initiate ERT.

p.M290I is a previously known but poorly described GLA mutation. To our knowledge, this is the first report of p.M290I mutation-associated disease activity that offers strong evidence of its pathogenicity.

## Introduction

Fabry disease (FD) [Online Mendelian Inheritance in Man® (OMIM®) entry # 301500] is an X-linked hereditary disease resulting from mutations in the GLA gene, leading to deficient activity of the enzyme alpha-galactosidase A (α-Gal A). The enzymatic defect results in progressive accumulation of undegraded glycosphingolipids in cell lysosomes, predominantly globotriaosylceramide (Gb3) and globotriaosylsphingosine (LysoGb3) [1,2]. Generally, nonsense, frame-shift, and splicing mutations result in the absence of enzyme protein or mutant enzymes with very low activity (less than 1%) [3]. These patients develop classic FD symptoms with the onset of acroparesthesias, angiokeratoma, hypohidrosis, and corneal and lenticular opacities during childhood or adolescence. Progressive proteinuria, glomerular filtration rate (GFR) decline, and tubular damage usually appear during the second or third decades of life, while end-stage renal disease (ESRD) develops during the fourth decade of life. Life-threatening injury to the kidneys, heart, and central nervous system occurs in later decades [4-6]. While males develop the classic full-blown disease, the females’ spectrum of phenotypes ranges from asymptomatic to severe as in males, depending on random X chromosome inactivation (lyonization) [7,8]. In contrast, non-classic, late-onset FD is associated with milder enzyme deficiency, mostly because of missense mutations. The residual α-Gal A activity determines a less severe phenotype, which usually lacks the childhood manifestations and develops exclusively with cardiac or renal manifestations [2,3,9]. Diagnosis of FD is made by measuring α-Gal A activity in plasma, leukocytes, dried blood spot (DBS), or fibroblasts, but should always be confirmed with genotype analysis. This is particularly important in suspected female patients since they can present normal enzymatic activity [2,10,11]. Enzyme replacement therapy (ERT) can improve the natural course of this disease by preventing the deposition of glycosphingolipids in the organs. Since an early diagnosis is important for treatment success, the detection of FD before the appearance of clinical manifestations is crucial [11,12].

## Case presentation

**Figure 1:**
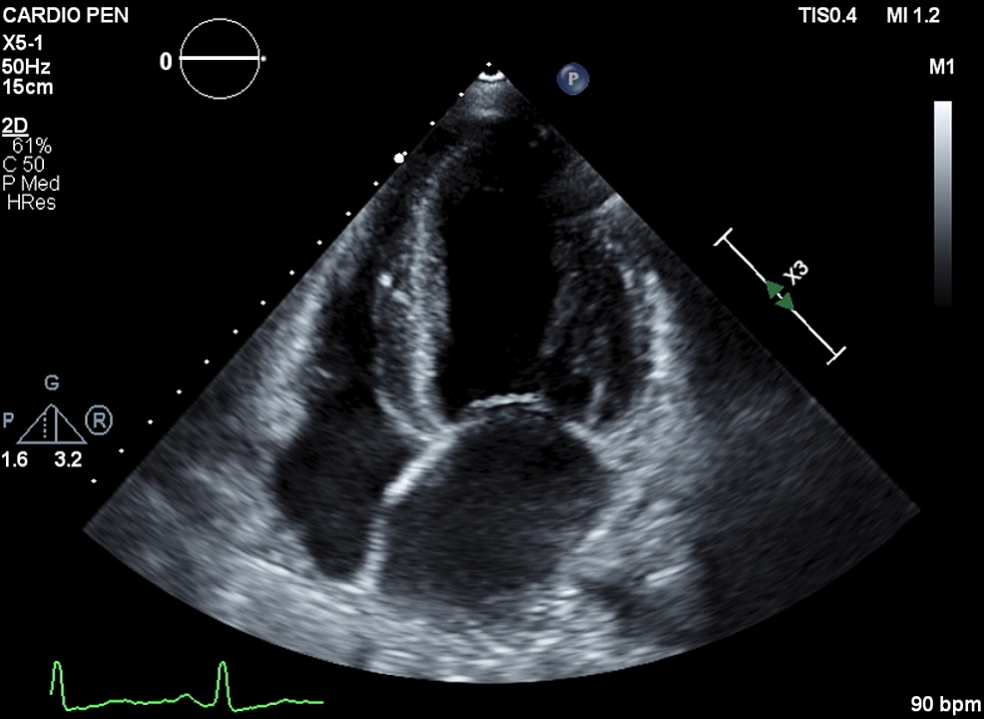
Echocardiography of the patient showing a slight left ventricular hypertrophy bpm: beats per minute

We describe the case of a 47-year-old female with an unremarkable past medical history except for migraines during her adolescence. At the age of 35, she had a preterm delivery secondary to preeclampsia and evolved with chronic proteinuric kidney disease in the puerperium. During the seven years that followed, despite receiving optimal medical treatment, the disease progressed to stage 5 chronic kidney disease (CKD), and renal replacement therapy (RRT) was started with peritoneal dialysis (PD). Five years later, in 2016, she was enrolled in a pilot screening study for FD, which included all patients undergoing RRT at our nephrology unit. Using fluorogenic methods, the enzymatic activity of α-Gal A was determined in DBS. The concentration of the biomarker LysoGb3 was also determined through high-performance liquid chromatography (HPLC) and tandem mass spectrometry. Sequence analysis of the GLA gene was carried out by polymerase chain reaction (PCR) multiplex, using genomic DNA samples. Genotype analysis was performed for all of the female subjects since heterozygous women cannot be reliably defined by enzymatic analysis alone. The subject’s genetic analysis revealed the presence of a heterozygous mutation c.870G>C (p.Met290Ile; M290I) in exon six of the GLA gene with a LysoGb3 level of 1.4 ng/mL (reference value: ≤ 1.8 ng/mL). The subsequent clinical investigation revealed the presence of hypohidrosis and heat intolerance, maintained proteinuria (albumin-to-creatinine ratio of around 1,000 mg/g), and slight left ventricular hypertrophy (LVH), with a left ventricle mass index (LVMi) measuring 108.5 g/m^2^ on echocardiography (Figure 1).

**Figure 2:**
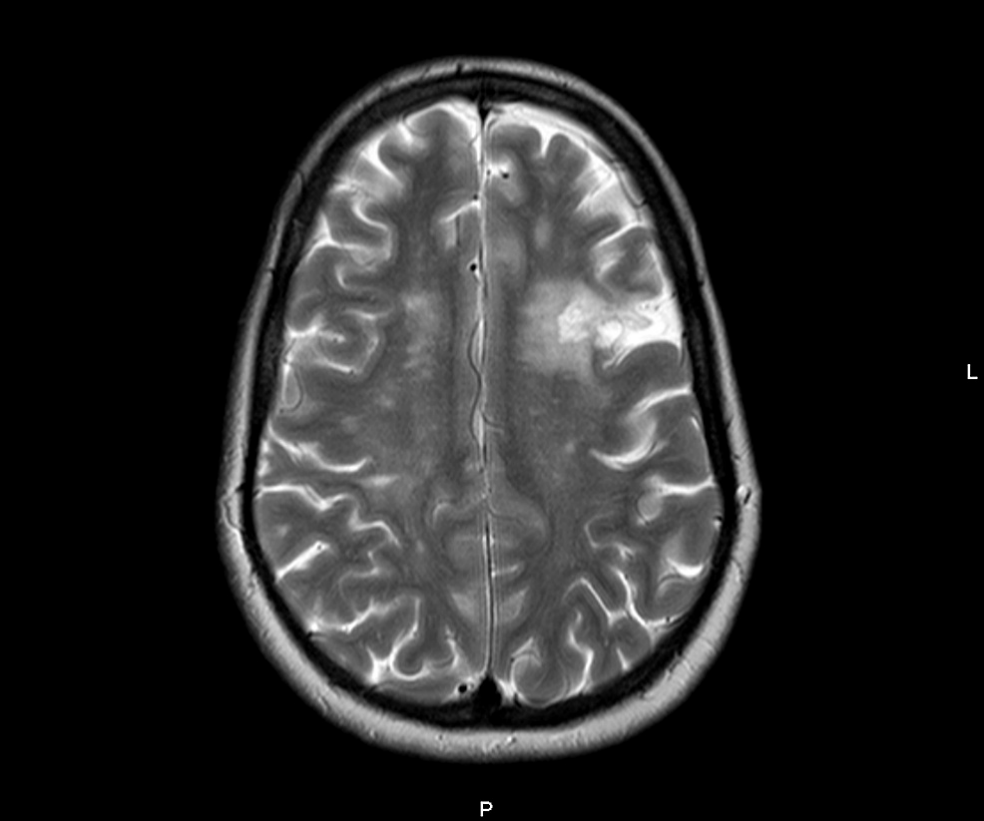
Cerebral magnetic resonance T2-weighted image showing a small recent frontoparietal ischemic lesion

Electrocardiographic (ECG) study, slit-lamp ophthalmological examination, dermatoscopy, audiogram, chest radiography, and respiratory function tests were unremarkable. The proband did not meet the criteria for a definitive FD diagnosis according to the European Fabry Working Group [13]; however, since the mutation was described as pathogenic and associated with a classic FD phenotype, the patient remained under follow-up at our nephrology metabolic diseases consultation. The family screening revealed the presence of this mutation in the proband's father and daughter. The progenitor was 73 years old, without any known past medical history as he had never sought medical treatment. The α-Gal A activity level was inferior at 2.8 µmol/l/h (reference value: ≥15.3 µmol/l/h), but he refused further investigation. Our patient's daughter was 14 years old and had been followed up for six years at a pediatric consultation due to generalized abdominal pain. The etiological investigation was inconclusive and the abdominal pain had improved spontaneously at the age of 11 years. After one year of follow-up, our patient received a cadaveric renal transplant, but the procedure was immediately complicated by a graft venous thrombosis and anastomosis hemorrhage. A panel of thrombophilia and coagulation tests were performed and no acquired or hereditary disease was found. Urgent graft removal was necessary, and the patient resumed PD. Later that same year, reassessment exams revealed maintenance of the mild LVH, but with an increase in the LVMi to 130 g/m^2^ on the echocardiogram (Video 1) and a small recent frontoparietal ischemic lesion on cerebral MRI (Figure 2).

**Video 1:** Echocardiography of the patient showing the worsening of left ventricular hypertrophy with elevated left ventricle mass index bpm: beats per minute

Furthermore, the LysoGb3 level, measured by the same laboratory and using the same method as before, increased to 29.2 ng/mL. These clinical changes led to the decision to initiate ERT, which the patient accepted.

## Discussion

M290I was reported by Shabbeer et al. in 2006 as a causal mutation of the classic form of FD [14]. Nevertheless, until the writing of this manuscript, there have been only a few descriptions of this genetic variant in the scientific literature. A Portuguese study analyzed a total of 11 FD patients and described two patients with p.M290I mutation, without detectable Gb3 accumulation. One was a 44-year-old female and the other a two-year-old male. However, in the latter, it should be noted that the Gb3 was not expected to be excreted in traceable amounts at such a young age [15]. Another study worth mentioning was published by Pan et al. in 2016, which was designed to evaluate the genotype-phenotype relationship in 73 Chinese FD patients. Clinical phenotype was classified into classic and atypical FD and the latter was further classified into renal-dominant FD and cardiac-dominant FD. Classic FD was diagnosed if patients met at least three of the following criteria: low activity of α-Gal A in leucocytes; symptom onset before the age of 25; the presence of a GLA gene variation in a patient who suffered from at least one of the typical FD symptoms (neuropathic pain, cornea verticillata, and clustered angiokeratoma) or having one or more family members with a definitive diagnosis of classical FD. Contrary to other reports, the p.M290I mutation was not associated with the classic FD phenotype [16]. Another Swiss investigation with a similar design analyzed 69 FD patients during their routine annual examinations. M290I mutant enzyme was found in a 48-year-old heterozygous female with a classic FD phenotype but with low serum LysoGb3 levels [17]. A Spanish newborn screening identified one male patient with FD and the p.M290I genetic variant but was unable to provide any information about the clinical expression of this mutation since the diagnosis was made between the third and fifth days of life [18]. The study describing the most number of patients carrying the M290I mutant enzyme is a Brazilian one; it screened a total of 25,223 dialysis patients. Among 89 FD-positive patients, the p.M290I mutation was present in 22 [19]. However, the authors did not provide detailed information about the clinical manifestations or α-Gal A activity and LysoGb3 levels of these patients. Finally, a recent Portuguese screening of 150 hypertrophic cardiomyopathy (HCM) patients found 25 patients with FD. Among these, there was a 43-year-old female who carried the GLA gene variant p.M290I, with a non-detectable LysoGb3 plasma level. Of note, this patient had asymmetrical LVH, proteinuria, and brain white matter lesions [20]. Despite the presence of clinical manifestations related to the classic FD presentation, none of the aforementioned scientific data provides evidence of lysosomal Gb3 and/or LysoGb3 accumulation associated with p.M290I mutation.

The case we reported thus differs from the others previously described. Overall, the kidney disease and the ischemic stroke observed in our patient cannot be attributed to FD beyond a reasonable doubt. At the time of the screening, in addition to renal involvement, our patient had only a slight LVH, which could easily be explained by the end-stage renal failure itself. The initial clinical presentation and the progression of CKD are not suggestive of FD nephropathy. Migraine, which the patient had suffered during her adolescence, is associated with preeclampsia, which is an independent risk factor for future stroke. Also, women with prior preeclampsia have increased white matter hyperintensities on brain MRI. Furthermore, patients with CKD, particularly those on regular HD treatment, are at a heightened risk of stroke. However, the LVH showed signs of progression which, combined with the rise in LysoGb3 levels, made the patient's FD activity evident, thereby encouraging us to initiate ERT.

## Conclusions

We described a case of FD due to a previously known but still poorly described GLA mutation and offered strong evidence of its pathogenicity. To our knowledge, this is the first report of p.M290I mutation-associated disease activity evidenced by elevated levels of serum LysoGb3.

In the absence of classic FD symptoms such as neuropathic pain, cornea verticillata, and angiokeratoma, and without a definitive diagnosis of the patients’ renal and cerebral ischemic disease, it is more likely that this mutation was associated with a late-onset FD phenotype. However, contrary to what the available data suggest, we reported an association between the p.M290I GLA mutation and considerable α-Gal A deficiency, high levels of substrate accumulation, and clinically significant disease. It is our opinion that the presence of this mutation warrants strict and consistent patient follow-up.
